# Evaluation of skin graft take following post-burn raw area in normovolaemic anaemia

**DOI:** 10.4103/0970-0358.59281

**Published:** 2009

**Authors:** Pawan Agarwal, Brijesh Prajapati, D. Sharma

**Affiliations:** Plastic Surgery Unit, Department of Surgery, N.S.C.B. Government Medical College, Jabalpur-482 003, MP, India

**Keywords:** Anaemia, skin graft take, wound healing

## Abstract

**Background:**

Traditional wisdom is that wound healing is directly related to haemoglobin level in the blood; therefore blood transfusion is given in anaemic patients to raise the haemoglobin level for better wound healing.

**Methods:**

Evaluation of wound healing in the form of split thickness skin graft take was done in 35 normovolaemic anaemic patients (haemoglobin level of < 10 gm/ dl) and compared with control group (patients with haemoglobin level of 10 or > 10 gm/ dl).

**Results:**

There was no statistically significant difference in mean graft take between the two groups.

**Conclusion:**

It is not mandatory to keep haemoglobin level at or >10 g/dL or PCV value at or >30% for skin graft take, as mild to moderate anaemia per se does not cause any deleterious effect on wound healing; provided perfusion is maintained by adequate circulatory volume. Prophylactic transfusion to increase the oxygen carrying capacity of the blood for the purpose of wound healing is not indicated in asymptomatic normovolemic anaemic patients (with haemoglobin levels greater than 6g/dL) without significant cardiovascular or pulmonary disease.

## INTRODUCTION

It is a well-known fact that sufficient oxygen supply and avoidance of wound infection is critical to the healing process as ischemic tissues heal poorly and get easily infected. Animal experimentations have shown that arterial hypoxia retards healing by reducing wound tensile strength, therefore anaemia seems to be detrimental to healing.[1] Previous theory in the last decade has been that haemoglobin level should be maintained above 10g/dL to promote wound healing.[2,3] As a result, some physicians and surgeons have been promoting blood transfusion to raise the haemoglobin level for better wound healing. The decision to transfuse blood is guided largely by the haemoglobin concentration of the patient, and as a rule if the patient's haemoglobin or haematocrit drops below 10g/dL or 30% respectively, prophylactic transfusions are usually administered. This empirical, dogmatic approach to blood transfusion was based on the concept that the risk-benefit ratio favoured transfusion in anaemic patients. Today, this principle no longer appears valid since it has become increasingly clear that blood transfusion, although potentially beneficial, is not without risk.[4] A strategy commonly employed by surgeons and anaesthesiologists is to request more units of blood than they anticipate transfusing intraoperatively to provide a margin of safety in the event of unexpected haemorrhage. The ready availability of one or two cross-matched units of blood in the operating room may even serve as a stimulus for unnecessary blood transfusions. It is important to re-evaluate the blood transfusion policy because of an inherent risk of transfusion reaction, transmission of many viral, bacterial and parasitic diseases, increased workload over blood banks and increased cost of patient care.

Blood transfusions have non-specific immuno-suppressive properties that render recipients susceptible to infectious complications and retard wound healing.[5,6] Other strategies to reduce intraoperative blood loss include the use of topical thrombin and epinephrine, extremity tourniquets, acute normovolaemic hemodilution, and hypotensive anaesthesia.[7] The present study was conducted to evaluate the state of wound healing *vis-à-vis* split skin graft take in normovolaemic anaemic patients.

## MATERIAL AND METHODS

Before starting the study, institutional ethical committee approval was taken. Seventy consecutive patients following post-burn raw area, due for split thickness grafting, were randomised prospectively irrespective of age, sex, weight and nutritional status. Patients with thermal burn >25% were excluded from the study. Patients with known history of diabetes mellitus, hypertension, nephritis, jaundice, bronchial asthma, tuberculosis, malignancy and history of steroid administration were also excluded. On admission, all the burn patients were treated with a standard regimen consist of fluid resuscitation, antibiotics, wound management and care of nutrition till their wound either healed or granulated. During this period blood transfusions if necessary were given. After the wound granulated no patients were given blood transfusion. Patients were divided into study groups (n = 35) with haemoglobin < 10 g/dL, haematocrit <30% and serum protein < 6 gm% and control group (n = 35) with haemoglobin level = or > 10 g/dL, haematocrit = or > 30% and serum protein = or >6 gm% [Tables 1, 2]. After debridement of recipient site, split thickness skin graft was harvested from thigh and applied on the raw area. The average raw area covered with skin graft in study group was 7% and in control group was 8%. Assessment of skin graft take was done on 10^th^ postoperative day.

**Table 1:** Mean Hb, PCV and serum protein in study group and control group

*Parameters*	*Study group*			*Control group*		
		
	*Mean Hb gm%*	*Mean PCV*	*Mean serum protein*	*Mean Hb gm%*	*Mean PCV*	*Mean serum protein*
Preoperative	8.5	25.0		10.92	32.0	
1^st^ postoperative	8.09	23.5	4.68	10.6	30.0	6.1
10^th^ postoperative	8.92	26.0		11.0	33.02	

**Table 2:** Mean thermal burn, raw area and graft take in both the groups

	*Mean thermal burn*	*Mean raw area*	*Graft take*
Study group	20%	7%	100% in 27/35 (77.14%)
			90-100% in 8 (22.8%)
Control group	22%	8%	100% in 29/ 35 (82.85%)
			90-100% in 6 (17.1%)

Haemoglobin and haematocrit were again measured on 1st and on 10th postoperative day. Results were assessed clinically by reviewers who were blinded to the haemoglobin and haematocrit levels of the patients. Statistical analysis was done using Chi Square test and level of significance was taken as <0.005. Blood transfusion was avoided and only colloids are administered when it became mandatory to make the patients hemodynamically stable.

## RESULTS

The youngest patient was five years old and the oldest was 60. The mean preoperative haemoglobin and haematocrit level in the study group was 8.5 g/dL and 25% respectively. The mean 1^st^ postoperative and 10^th^ postoperative day haemoglobin/ haematocrit levels in the study group were 8.09 g/dL/23% and 8.92 g/dL/26% respectively [Table 1]. The mean preoperative haemoglobin and haematocrit level of control group was 11.02 g/dL and 32%. The mean 1^st^ post-operative and 10 postoperative day haemoglobin/ haematocrit levels were 10.8 g/dL /31% and 11.5 g/dL /34.02% respectively in the control group [Table 1]. The mean serum protein in the study group was 4.68 gm% with mean serum albumin and globulin was 2.5 gm% and 2.1 gm%. In the control group, mean serum protein was 6.1gm% with serum albumin and serum globulin level 3.6 gm% and 2.5 gm% respectively. Evaluation of graft-take done on the 10^th^ postoperative day revealed 100% graft take in 27/ 35 (77.14%) patients among study group and 29/ 35 (82.85%) patients among control group. 90-100% graft take was observed in 8 (22.8%) patients among study group and in 6 (17.1%) patients among control group [Table 2]. None of these patients required secondary surgery for their loss of graft and all healed spontaneously by conservative treatment. Graft take was almost in equal proportion in study and control group. Statistically there was no difference in the skin graft take in both groups (P> 0.01).

## DISCUSSION

Various factors like anaemia, hypoproteinemia, infection, avitaminosis and prolonged use of steroids are known to cause poor wound healing; anaemia is frequently blamed because the haemoglobin is considered essential to maintain proper oxygenation.[8] Elective surgery is usually delayed to combat this deficiency, either by preoperative haematinics or by blood transfusions. The effect of anaemia on wound healing remains uncertain and the decision to transfuse blood is influenced by the 10/30 empirical dictum that a patient with haemoglobin level < 10 gm% and haematocrit level < 30% requires blood transfusion. Despite various human and animal studies, no clinical consensus has been achieved on the absolute threshold for prophylactic transfusion in anaemic patients. However, studies on the physiology of anaemia have demonstrated that weakness at rest rarely occurs until haemoglobin level falls below 6g/dL and cardiovascular failure does not occur in normovolaemic, anaemic individuals until haemoglobin level falls below 3-4 g/dL or haematocrit drops below 10-12%.[4] Arterial blood carries 0_2_ as oxy- haemoglobin in the red blood cells which, when exposed to low tension of oxygen in the wounded tissues, dissociates and oxygen leaves the blood stream and enters into the tissues. In the wounded tissue increased CO_2_ tension, decreased O_2_ tension, decreased velocity of blood, vasodilatation and increased temperature favour the dissociation of oxy-haemoglobin thus supplying more oxygen to the wounded tissue. Furthermore, in anaemia there is increased cardiac output, decreased blood viscosity and decreased peripheral resistance; all of which lead to increased perfusion thereby mitigating the ill effects of anaemia.[9,10] As wounds consume less O_2_ in comparison to normal tissues, P0_2_ in wounded tissue can be maintained by increased perfusion with anaemic arterial blood despite its low oxygen content. Oxygen tension in the tissues depends upon diffusion across wound capillaries therefore the partial pressure of oxygen in the arterial blood (PaO_2_) is a more important determinant for delivering oxygen in the tissues than the arterial oxygen content.[11–13]

In animal experimentation, haemoglobin as low as 2-3g/dL and PCV below 15 are compatible with normal development of tensile strength provided blood volume remains normal.[14] This value seems to be very low for humans, therefore various investigators have tried to find out the minimal tolerable limit of haemoglobin below which wound healing will be impaired and blood transfusion will be necessary. Haemoglobin mass of an average 70 kg man is capable of delivering 1000ml of oxygen/minute. Since, oxygen-haemoglobin dissociation curve permits only 60% delivery of haemoglobin bound oxygen at a partial pressure of greater than 20 mm of Hg. it is possible for a haemoglobin concentration of 6.5 g/dL to deliver the required 260 ml of oxygen/minute. Further, there is increase in concentration of 2, 3 diphosphoglycerate (2, 3 DPG) in red blood cells which favours the unloading of oxygen from haemoglobin to erythrocytes.[14] These normal physiological responses to anaemia tend to restore the normal oxygen delivery to the tissues, but common sense dictates that at some point the oxygen carrying capacity is no longer sufficient to maintain PO_2_ in the capillaries and in the tissues then that level of anaemia would be detrimental for wound healing. This critical point is defined by various investigators in the range of haemoglobin 6 g/dL and PCV 18% and this point is considered as the transfusion trigger.[15]

In our study, equal acceptance of graft in study and control group implies that anaemia and hypoproteinemia do not necessarily retard wound healing. In this study six (17.1%) patients in the study group and four (11.4%) in the control group were given colloids to maintain their blood volume during operation. None of the patients required blood transfusion during surgery or postoperative period. All the patients in the study group tolerated anaesthesia well and remained stable throughout the procedure. This shows that perioperative blood transfusion can be avoided in the surgical care of most patients who have normovolaemic anaemia without mortality and without significant morbidity.[16]

Interpolation of these data to surgical patients requires caution as coexisting cardiovascular or pulmonary disease may impair the compensatory response to anaemia. Moreover, increased metabolic activities and presence of fever increase the oxygen demand that may exceed the ability of the compensatory response, risking oxygen deprivation in vital organs. These data are specific for skin and subcutaneous wound healing and internal wound healing e.g. gastrointestinal anastomosis are different from the skin and subcutaneous tissue healing.[17]

We have used World Health Organisation (WHO) classification to categorize our patients. According to WHO classification anaemia is classified as mild when haemoglobin is between 9-11 gm%, moderate when haemoglobin is 7-9 gm% and severe in cases of haemoglobin less than 7 gm%.[18]

## CONCLUSION

It is not mandatory to keep haemoglobin level at or >10 g/dL or PCV value at or >30% for skin graft take, as mild to moderate anaemia *per se* does not cause any deleterious effect on wound healing; provided perfusion is maintained by adequate circulatory volume. Prophylactic transfusion to increase the oxygen carrying capacity of the blood for the purpose of wound healing is not indicated in asymptomatic anaemic patients (with haemoglobin levels greater than 6g/dL) without significant cardiovascular or pulmonary disease. However, severe anaemia (haemoglobin 6g/dL) needs to be corrected before surgery. In a patient with significant cardiovascular or pulmonary disease, transfusion policy must be individualized and should be based on evidence of supply dependent oxygen consumption. Avoiding unnecessary transfusions in this era of heightened awareness of their risks appears beneficial.
